# Biochemical and structural characterization of AmyY from *Alkalimonas* sp. NCh-2 reveals a class of metal-free α-amylases in glycoside hydrolase family 13

**DOI:** 10.1128/aem.00660-26

**Published:** 2026-08-12

**Authors:** Fang Zhao, Ting-Ting Xu, Chun-Mei Yu, Jun-Mei Ding, Bi-Hong Zhou, Yu-Zhong Zhang, Xiu-Lan Chen, Yi Zhang, Yu-Qiang Zhang, Mei-Ling Sun

**Affiliations:** 1 Ministry of Education Key Laboratory of Evolution and Marine Biodiversity, Frontiers Science Center for Deep Ocean Multispheres and Earth System, Shandong Provincial Key Laboratory of Microbial Resource Exploration and Innovative Utilization & College of Marine Life Sciences, Ocean University of China 12591, Qingdao, China; 2 Laboratory for Marine Biology and Biotechnology, Qingdao Marine Science and Technology Center, Qingdao, China; 3 Marine Biotechnology Research Center, State Key Laboratory of Microbial Technology, Shandong University 12589, Qingdao, China; 4 Engineering Research Center for Valorization of Unique Bio-Resources in Yunnan, Ministry of Education, Yunnan Normal University 66343, Kunming, China; 5 Guangzhou Blue Moon Industrial Co., Ltd, Guangzhou, China; University of Illinois Urbana-Champaign, Urbana, Illinois, USA

**Keywords:** alkaline α-amylase, structure, metal-free, truncation, thermostability

## Abstract

α-Amylases (EC 3.2.1.1) are among the most important industrial enzymes in starch saccharification, detergent, paper, and textile industries. Canonical α-amylases typically contain Ca^2+^ in their structure, which is not directly involved in catalysis but essential in maintaining the structural integrity of α-amylases. Ca^2+^ ions are easily removed by chelating reagents, which may affect the activity and stability of α-amylases and thereby hinder their applications in some industries. Here, we identified a metal-free α-amylase, AmyY, from the bacterium *Alkalimonas* sp. NCh-2 isolated from an alkaline hot spring. AmyY contains a catalytic module of glycoside hydrolase family 13 (GH13) and a carbohydrate-binding module of family 20 (CBM20). AmyY exhibits high activity under alkaline conditions (pH 8.0–11.0) and tolerance to high salinity, chelating reagents, and surfactants. We solved the structures of AmyY and its complex with acarbose. Although the overall topology and active sites of AmyY resemble those of reported GH13 α-amylases, the structure of AmyY is devoid of metal ions. The inability of AmyY to bind metal ions results from replacements of acidic amino acid residues by neutral ones. Combined with biochemical, structural, and bioinformatic data, we demonstrated that AmyY and its homologs represent a class of metal-free α-amylases in GH13. Moreover, structural analyses revealed that the CBM20 of AmyY is highly flexible, and we obtained a mutant with enhanced thermostability by truncating the CBM20. This mutant demonstrates remarkable wash performance and desizing capability at alkaline pH, which underscores its application potential in the detergent and textile industries.

**IMPORTANCE:**

While canonical GH13 α-amylases typically contain Ca^2+^, our biochemical and structural data reveal that AmyY has evolved a metal-free architecture. This is achieved through the replacement of acidic metal-coordinating residues by neutral ones, abolishing metal binding without compromising catalytic efficiency. This work expands the structural and functional diversity of GH13 by defining a class of metal-free α-amylases, with AmyY and its homologs as representatives. Furthermore, the engineered AmyY with enhanced thermostability offers a candidate biocatalyst for industrial applications, such as the detergent industry. The structure of AmyY also offers a blueprint for engineering other α-amylases into metal-independent forms.

## INTRODUCTION

**Fig 1:**
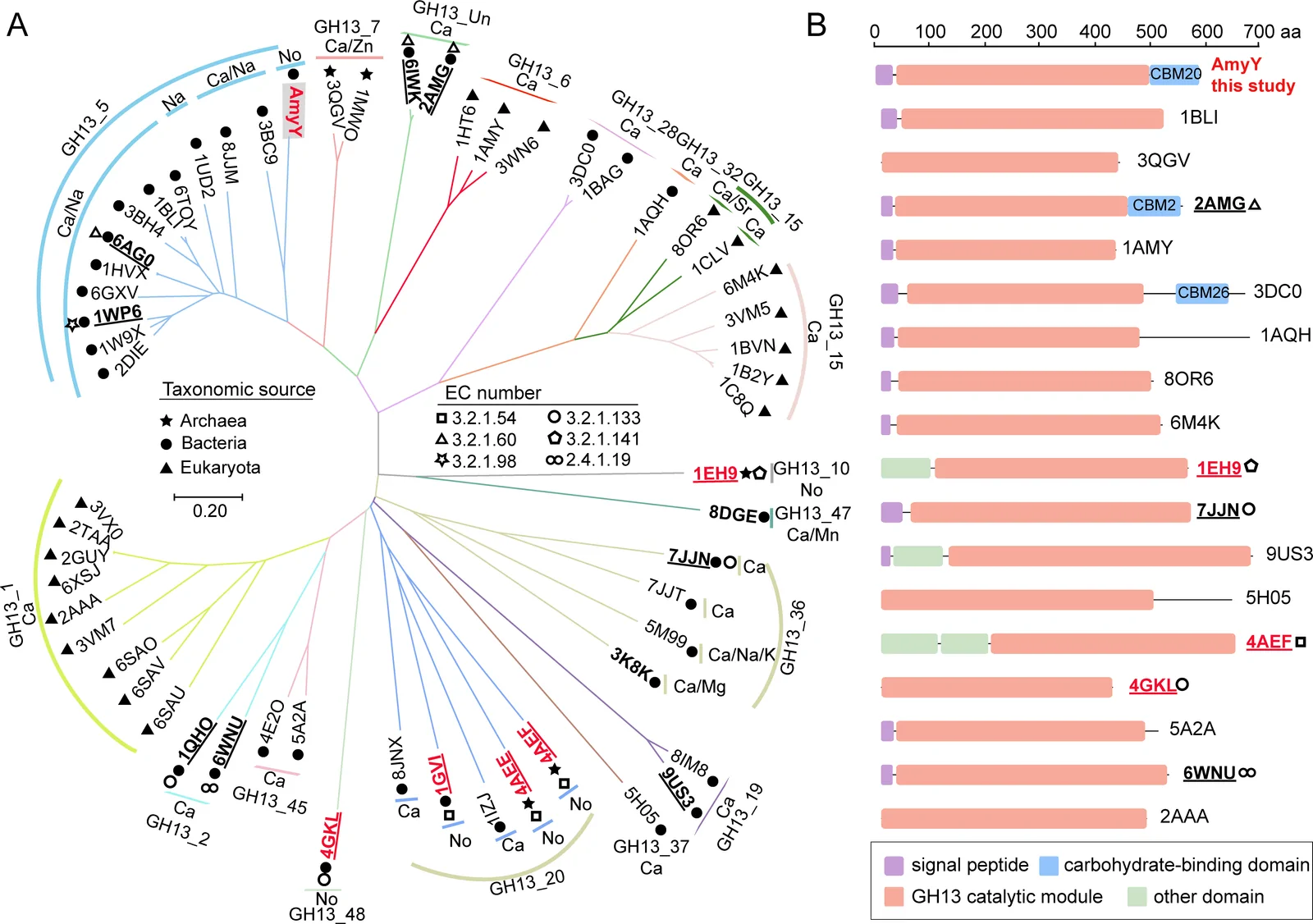
Phylogenetic analysis and domain architectures of structure-determined GH13 α-amylases. (**A**) Phylogenetic analysis of AmyY and other structure-determined α-amylases from different subfamilies of GH13. The tree was built by the neighbor-joining method with the Poisson model. A bootstrap test (1,000 replicates) was conducted. There was a total of 696 positions in the final dataset. Taxa are labeled with the PDB ID of α-amylases. Subfamily classifications and the presence of metal ions in each structure are indicated. (**B**) Domain architectures of AmyY and representatives from different subfamilies of GH13. Metal-free α-amylases are shown in red. Atypical α-amylases are highlighted in bold and underlined, and their EC (enzyme commission) numbers are indicated. EC 3.2.1.54, cyclomaltodextrinase; EC 3.2.1.60, maltotetraose-forming amylase; EC 3.2.1.98, maltohexaose-forming amylase; EC 3.2.1.133, maltogenic α-amylase; EC 3.2.1.141, maltooligosyltrehalose trehalohydrolase; cyclomaltodextrin glucanotransferase.

As good alternatives to replace harmful chemical products, enzymes are widely used in various industries. α-Amylases (EC 3.2.1.1), which randomly cleave the α-1,4-glucosidic linkages in starch, are among the most important industrial enzymes and represent approximately 25% of the enzyme market (1, 2). As classified in the Carbohydrate Active Enzymes (CAZy) database (http://www.cazy.org) (3), the vast majority of α-amylases belong to glycoside hydrolase (GH) family 13 that is subdivided into 47 subfamilies (4). GH13 α-amylases share a catalytic module that is composed of three domains, domain A with a conserved (β/α)_8_ TIM (triosephosphate isomerase)-barrel fold, domain B inserted within domain A, and domain C containing a Greek key motif (5). Sometimes, additional domains, such as carbohydrate-binding modules (CBMs), occur at the C-terminal or N-terminal (6) (Fig. 1). Three conserved catalytic residues (Asp, Glu, and Asp) that form the active site are located at the interface between domains A and B (2).

GH13 α-amylases, regardless of their origin (archaea, bacteria, or eukaryota), typically contain Ca^2+^ ions in their structures (Fig. 1A). While Ca^2+^ ions are not directly involved in catalysis, they are functionally critical in maintaining the structural integrity and thereby the activity and stability of α-amylases (7). In the starch industry, reactions of α-amylases generally require the addition of Ca^2+^. However, Ca^2+^ inhibits the activity of glucoamylases and glucose isomerases that are used in subsequent starch-processing reactions (8, 9). Moreover, the addition of Ca^2+^ accelerates the formation of calcium oxalate, a substance that may block process pipes and heat exchangers (10). In the detergent industry where chelating reagents are usually additives of detergents, Ca^2+^ ions are easily removed by chelating reagents, which severely limits the use of α-amylases (11). Although Ca²^+^ is typically essential for α-amylase structure, several exceptions have been reported. For instance, the α-amylase AmyK38 (PDB: 1UD2) is Ca²^+^-free and contains only Na^+^, which displays catalytic activity only when Na^+^ is present (11, 12). Other atypical metal-free α-amylases include three cyclomaltodextrinases (EC 3.2.1.54; PDB: 4AEF, 4AEE, and 1GVI) (13–15), a maltooligosyltrehalose trehalohydrolase (EC 3.2.1.141; PDB: 1EH9) (16), and a maltogenic α-amylase with nonreducing end-attacking activity (EC 3.2.1.133; PDB: 4GKL) (17). Although the atypical α-amylases retain the conserved GH13 catalytic residues (Asp, Glu, and Asp) and catalytic mechanism for hydrolysis, their substrate-binding regions differ from those of typical α-amylases, and they do not preferentially act on starch (13–17). To date, no typical GH13 α-amylase has been experimentally determined to be metal-free.

Alkaline α-amylases display high catalytic activity and stability under alkaline conditions (pH > 8.0) (18). Alkalophilic bacteria are good producers of alkaline α-amylases. To date, many alkaline α-amylases of bacterial sources have been reported (18–22). These α-amylases possess application advantages in starch saccharification, detergent, paper, and textile industries. The alkaline α-amylase Termamyl (Novozymes), produced by a genetically modified *Bacillus licheniformis*, has been commercialized and widely used as a detergent additive. In addition, to improve enzymatic performances, such as catalytic activity, thermostability, and oxidative stability, some alkaline α-amylases have been engineered by directed evolution or rational design (23–26). Despite these studies, because of the industrial importance of alkaline α-amylases, there is ongoing interest in exploring novel alkaline α-amylases, such as thermostable and Ca^2+^-independent ones.

In this study, we report a novel alkaline α-amylase, AmyY, which represents a class of metal-free α-amylases in GH13. AmyY, containing a GH13 catalytic module and a CBM of family 20 (CBM20), was from *Alkalimonas* sp. NCh-2 that was isolated from an alkaline hot spring. We biochemically characterized AmyY and demonstrated that AmyY is a highly active alkaline α-amylase with high resistance against salinity, surfactants, and chelating reagents. Crystal structures of AmyY and its complex with acarbose were then solved. Combined with biochemical, structural, and bioinformatic data, we demonstrated that AmyY and its homologs represent a class of metal-free α-amylases, the inability of which to bind metal ions results from replacements of acidic amino acid residues by neutral ones. We also truncated the CBM20 of AmyY and obtained a mutant with enhanced thermostability, which demonstrated remarkable wash performance and desizing capability under alkaline conditions.

## RESULTS

### Sequence analysis of AmyY

**Fig 2:**
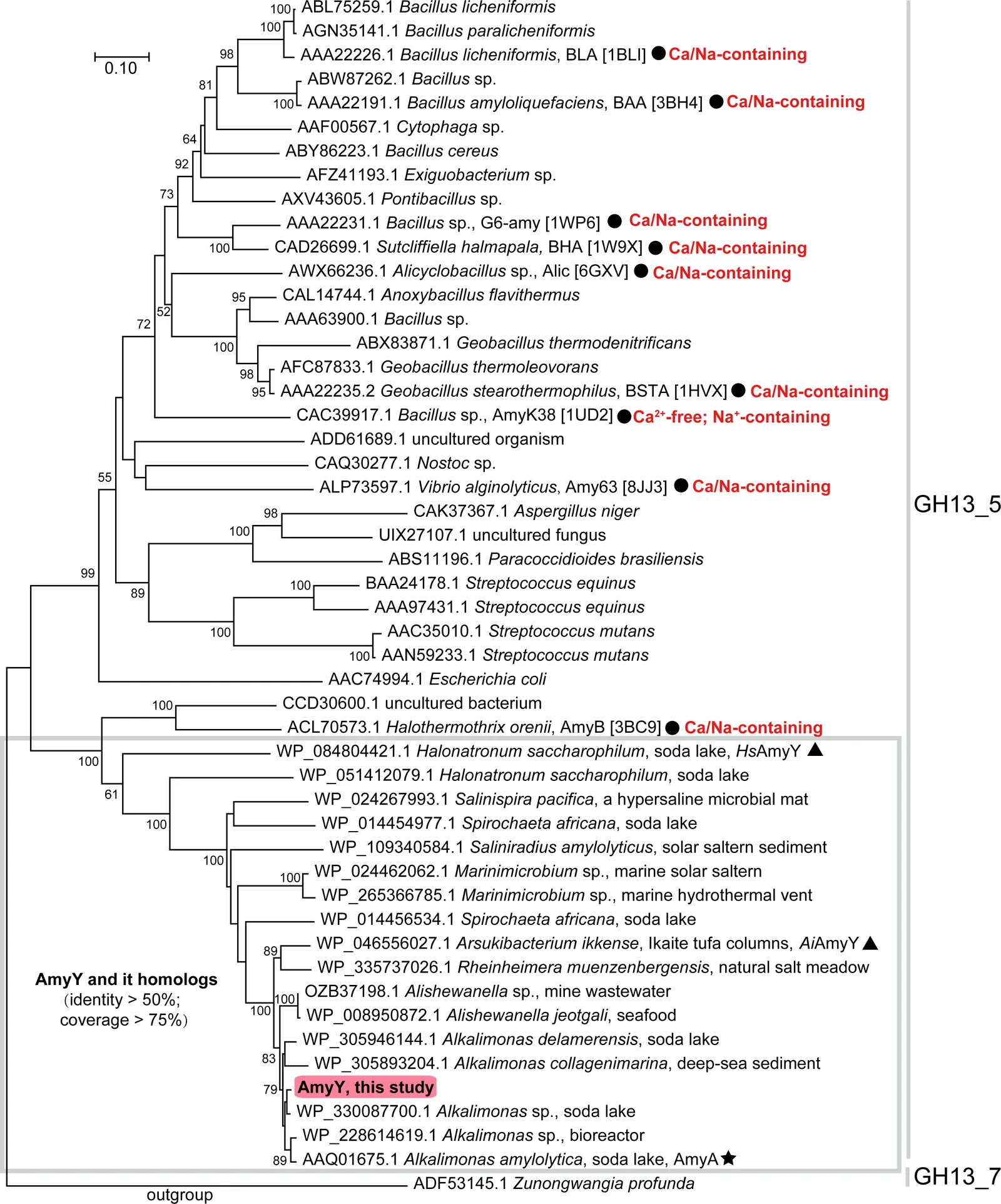
Phylogenetic analysis of AmyY and other α-amylases from GH13_5. The tree was built by the neighbor-joining method with the Poisson model using an α-amylase from GH13_7 as the outgroup. A bootstrap test (1,000 replicates) was conducted, and values above 50% are shown. There were a total of 518 positions in the final data set. Solid circles indicate reported structure-determined α-amylases, whose accession numbers in PDB are given in brackets. All these α-amylases contain metal-binding sites in their structures. The source strains and their isolation environments for AmyY homologs are provided. Solid triangles indicate the homologs of AmyY characterized in this study. The solid pentangle indicates the closest homolog of AmyY that has been biochemically characterized.

*Alkalimonas* sp. NCh-2 was isolated from a water sample collected from an alkaline hot spring in Xiangyun County, Yunnan Province, China, with soluble starch as the sole carbon source at 37°C and pH 10.0. In the genome of *Alkalimonas* sp. NCh-2 (GenBank: GCA_039631345.1), AmyY (GenBank: MEN3159421.1) was annotated as an α-amylase. According to the predicted results from the NCBI Conserved Domain Database (CDD) and SignalP 5.0, AmyY contains a 31-residue signal peptide, an N-terminal GH13 catalytic module, and a C-terminal CBM20 (Fig. 1B). Phylogenetic analysis showed that AmyY belongs to subfamily 5 of GH13 (GH13_5) (Fig. 1 and Fig. 2). It shares a high sequence identity of 95.1% (coverage: 100%) with AmyA, an alkaline α-amylase from *Alkalimonas amylolytica* (22). Among those structure-determined proteins in the Protein Data Bank (PDB), AmyY shows the highest sequence identity of 46.3% (coverage: 79%) to the α-amylase AmyB from *Halothermothrix orenii* (PDB ID: 3BC9) (5). In addition, AmyY homologs were retrieved from the NCBI non-redundant (nr) database with cut-off values of sequence identity >50% and coverage >75% (Fig. 2). A total of 17 AmyY homologs were retrieved, which are mainly from alkaline and hypersaline environments, such as soda lakes. Within GH13_5, AmyY and its homologs form a separate group.

### Biochemical characterization of AmyY

**Fig 3:**
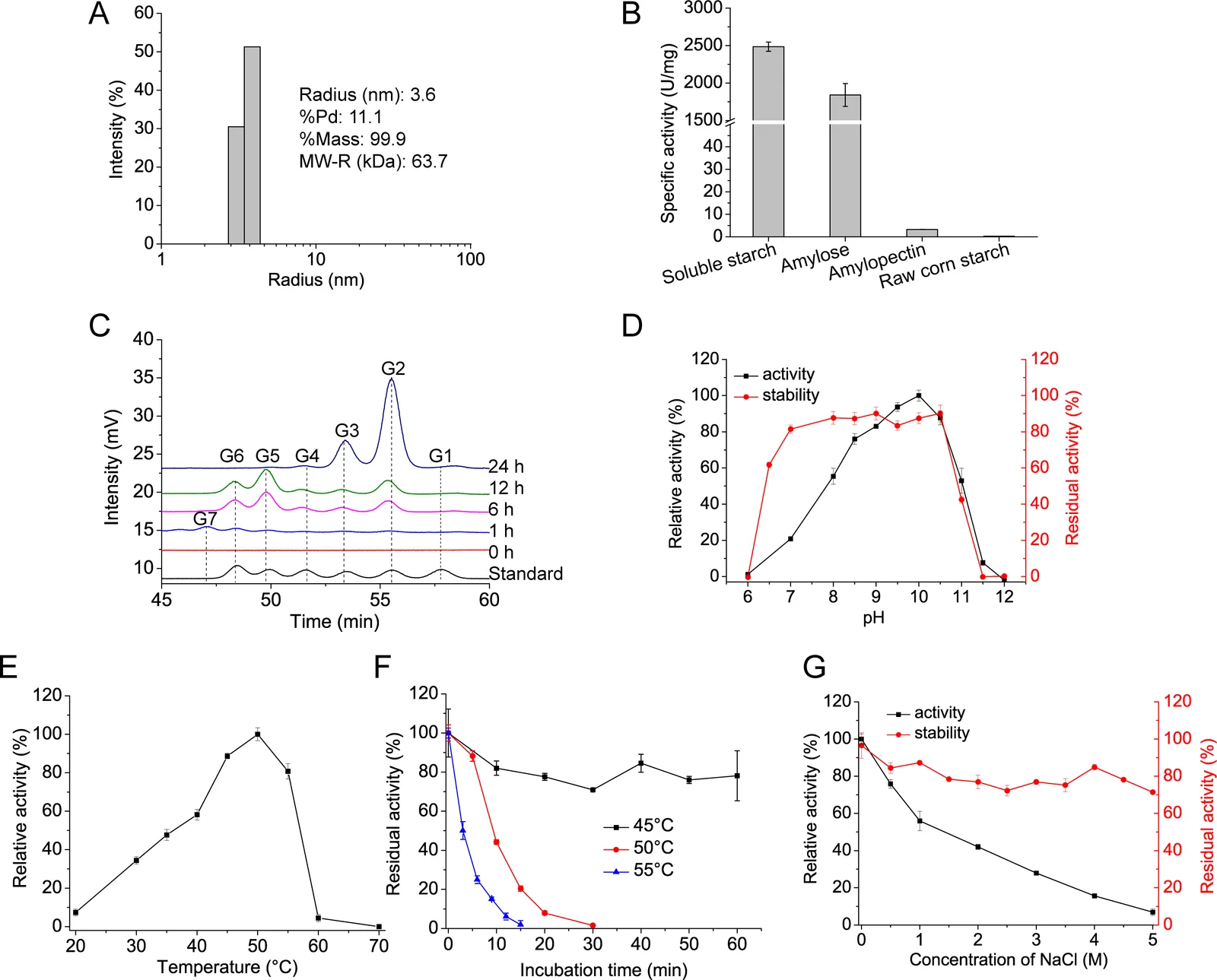
Biochemical characterization of AmyY. (**A**) DLS analysis of AmyY. (**B**) The substrate specificity of AmyY. Enzyme activities were determined at 50°C and pH 10.0. (**C**) A time-course degradation of soluble starch by AmyY. Soluble starch (270 µl; 1 mg/mL) and AmyY (30 µl; 50 µg/mL) were incubated at 30°C and pH 10.0. Reactions were terminated at 0, 1, 6, 12, and 24 h, separately, and the products were analyzed by HPLC. A mixture of glucose (G1) and malto-oligosaccharides (from G2 to G6) was used as the standard. (**D**) Effect of pH on the activity and stability of AmyY. For pH stability, AmyY was incubated in buffers from pH 6.0 to 12.0 at 40°C for 3 h, and residual activities were determined at 50°C and pH 10.0. (**E**) Effect of temperature on the activity of AmyY. (**F**) Effect of temperature on the stability of AmyY. AmyY was incubated separately at 45°C, 50°C, and 55°C for 1 h. At an interval of 10 min, residual activities were determined at 50°C and pH 10.0. (**G**) Effect of NaCl on the activity and stability of AmyY. For stability, AmyY was incubated in buffers containing 0–5.0 M NaCl at 4°C for 24 h. The residual activity was determined at 50°C and pH 10.0. Data shown in the graphs are from triplicate experiments (means ± standard deviations [SD] or representative results).

AmyY was overexpressed in *Escherichia coli* and purified by Ni-affinity chromatography. SDS-PAGE analysis showed that AmyY displayed an apparent molecular mass of approximately 62.8 kDa (data not shown), consistent with its calculated molecular mass of 63.7 kDa. This result indicated that the full-length AmyY, containing both its catalytic module and CBM20, was successfully produced in *E. coli*. Dynamic light scattering (DLS) analysis indicated that AmyY exists in a monomeric state in solution (Fig. 3A). AmyY displayed the highest activity on soluble starch, with a specific activity of 2,484.8 ± 61.6 U/mg (Fig. 3B). This value is higher than those for many other reported alkaline α-amylases (~5.0–1,300.0 U/mg) (27–31). AmyY also showed activity on amylose (1,840.6 ± 152.4 U/mg) but less than 0.1% activity on amylopectin and raw corn starch relative to that on soluble starch. Time-course treatment of soluble starch by AmyY showed that long-chain maltooligosaccharides (DP [degree of polymerization] values >6) were released at 1 h. Then at 12 and 24 h, maltopentaose (G5), maltohexaose (G6), and maltose (G2) were predominant, with G2 and maltotriose (G3) as the final products (Fig. 3C). The result suggested that AmyY is a canonical endo-acting α-amylase. AmyY displayed the maximum activity at pH 10.0 and retained >80% activity after 3-h incubation in 50 mM Britton-Robinson buffer at pH 8.0–10.5 and 40°C (Fig. 3D), indicating that AmyY is alkaliphilic. AmyY exhibited the highest activity at 50°C (Fig. 3E) and retained >80% activity after 1-h incubation at 45°C but lost almost all activity after incubation at 50°C for 30 min or at 55°C for 15 min (Fig. 3F), indicating that AmyY is moderately thermostable (32). AmyY is a halotolerant enzyme, retaining >70% activity after 24-h incubation in 5.0 M NaCl, although adding 0.5 to 5.0 M NaCl decreased the activity of AmyY (Fig. 3G). Mn^2+^ (10 mM) reduced the activity of AmyY by ~90% (Table 1), possibly due to non-specific binding that disrupts the active site or overall structure of AmyY, which awaits further investigation. Other metal ions, including K^+^, Li^+^, Ca^2+^, Mg^2+^, Co^2+^, Cu,^2+^ and Zn^2+^ (10 mM each), had little effect on the activity of AmyY. AmyY was quite stable in chelating reagents EDTA, EGTA, and sodium citrate, and surfactants SDS, Tween 20, and Triton X-100 at concentrations up to 100 mM (Table 1). Taken together, our results suggested that AmyY is an alkaline α-amylase with high activity, stability, and tolerance to high salinity, chelating reagents, and surfactants.

**TABLE 1:** Effects of metal ions and chemical reagents on the activity of AmyY

Metal ion or chemical reagent	Concentration	Relative activity (%)^*a*^
Metal ion		
K^+^	10 mM	99.4 ± 6.5
Li^+^	10 mM	101.2 ± 1.3
Ca^2+^	10 mM	102.7 ± 5.1
Mg^2+^	10 mM	94.3 ± 2.1
Co^2+^	10 mM	95.1 ± 11.8
Mn^2+^	10 mM	10.6 ± 0.9
Cu^2+^	10 mM	96.4 ± 3.1
Zn^2+^	10 mM	94.5 ± 3.3
Chelating reagent		
EDTA	100 mM	99.8 ± 5.6
EGTA	100 mM	98.7 ± 4.2
Sodium citrate	100 mM	106.2 ± 5.4
Surfactant		
SDS	100 mM	107.4 ± 4.2
Tween 20	100 mM	126.3 ± 4.6
Triton X-100	100 mM	125.1 ± 5.3

^*a*^ AmyY was pre-incubated in glycine-NaOH buffer (50 mM, pH 10.0) containing one of the metal ions and chemical reagents at 4°C for 1 h, and then the residual activities were determined in standard reaction mixture at 50°C and pH 10.0. The activity of AmyY incubated without any metal ion or chemical regent was taken as 100%. The data are from triplicate experiments (means ± SD).

### AmyY is metal-free

GH13 α-amylases typically contain Ca^2+^ ions in their structures (Fig. 1). The activity of α-amylases is frequently dependent on exogenous Ca^2+^ and inhibited by EDTA (5, 11, 12, 29). In contrast, AmyY displayed its full activity without any exogenous metal ions and showed high tolerance to chelating reagents (Table 1), which aroused our interest in whether AmyY contains Ca^2+^ ions. Therefore, metal ions in AmyY were determined by inductively coupled plasma-mass spectrometry (ICP-MS). To eliminate the effect of exogenous metal ions on ICP-MS data, a metal-free buffer, 50 mM Tris-HCl (pH 8.0), was used in protein purification. The results showed that the molar ratios of Ca^2+^, Na^+^, Li^+^, K^+^, Mg^2+^, Fe^2+^, Cu^2+^, and Zn^2+^ (metal cations frequently observed in protein structures) to AmyY were all below 0.15, suggesting that AmyY may not contain any of them.

### Overall structure of AmyY

The crystal structure of AmyY was determined to be 1.50 Å resolution (Table 2). The crystals of AmyY belong to the *P*2_1_2_1_2_1_ space group, with one AmyY molecule per asymmetric unit. The structure includes only the GH13_5 catalytic module (residues 32–493) and the electron density maps corresponding to CBM20 (residues 494–587) are not visible, likely because the CBM20 is conformationally unstable.

**TABLE 2:** Diffraction data and refinement statistics of AmyY and its complex with acarbose

Parameter	Value(s)^*a*^ for	
	AmyY	AmyY-acarbose
Space group	*P*2_1_2_1_2_1_	*P*3_1_21
a, b, c (Å)	49.44, 71.37, 146.43	140.20, 140.20, 84.03
α, β, γ (°)	90, 90, 90	90, 90, 120
Wavelength (Å)	0.9791	0.9791
Resolution (Å)	49.44-1.50 (1.52-1.50)	70.1-2.10 (2.16-2.10)
Redundancy	8.9 (5.9)	15.2 (13.5)
Completeness (%)	99.00 (93.30)	100.00 (100.00)
CC1/2	0.993 (0.969)	0.999 (0.964)
*R*_merge_ *^b^*	0.116 (0.163)	0.113 (0.755)
I/Sigma	14.3 (7.3)	17.5 (3.5)
*R*_work_	0.149 (0.152)	0.189 (0.265)
*R*_free_	0.172 (0.187)	0.227 (0.317)
Average B-factor	13.41	34.97
Macromolecules	10.92	35.01
Solvent	25.30	35.09
Ligands	21.46	31.49
Bond lengths (Å)	0.005	0.007
Bond angles (Å)	1.03	0.89
Ramachandran plot		
Favored (%)	97.61	97.39
Allowed (%)	2.39	2.61
PDB ID	24ZP	24ZQ

^*a*^ Numbers in parentheses refer to data in the highest resolution shell.

^*b*^ *R*_merge_ = Σ_hkl_Σ_i_|*I*(*hkl*)_i_ - <*I*(*hkl*)>|/Σ_hkl_Σ_i_ < *I*(*hkl*)_i_>, where* I* is the observed intensity, <*I*(*hkl*)> represents the average intensity, and *I*(*hkl*)*_i_* represents the observed intensity of each unique reflection.

**Fig 4:**
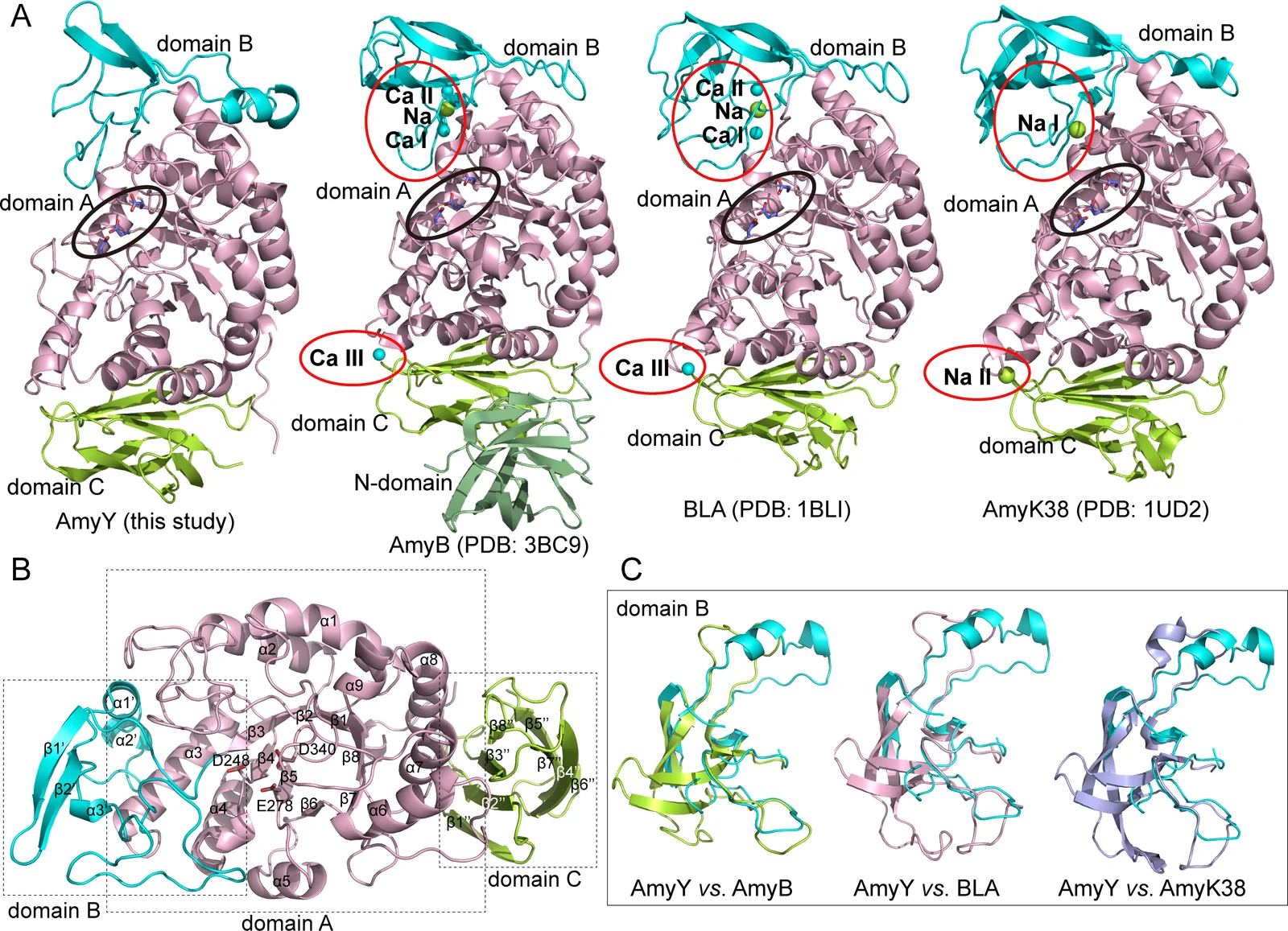
Overall structure of AmyY. (**A**) Comparison of the overall topologies of AmyY with reported α-amylases AmyB, BLA, and AmyK38. Domain A is colored in light pink, domain B in cyan, and domain C in lemon. Na^+^ and Ca^2+^ are shown as lemon and cyan spheres, respectively. The conserved catalytic residues (Asp, Glu, and Asp) are shown as purple sticks. Metal-binding sites and the catalytic center are highlighted, respectively, with red and black ellipses. (**B**) Overall cartoon view of AmyY. Catalytic residues Asp248, Glu278, and Asp340 are shown as sticks. (**C**) Structural superposition of the domain B of AmyY (cyan) with those of AmyB (left; lemon), BLA (middle; light pink), and AmyK38 (right; light blue).

The overall structure of AmyY exhibits the typical topology of GH13 α-amylases, consisting of domains A, B, and C (Fig. 4A). Domain A (residues 32–143 and 223–401) has a (β/α)_8_ TIM-barrel fold composed of eight peripheral β-sheets (β1–β8) and eight interior α-helices (α1–α8) (Fig. 4B), the most common protein fold (33). Domain B (residues 144–222) is inserted between the β3 and α3 of domain A, mainly composed of two anti-parallel β-sheets (β1′ and β2′). Domain C (residues 402–493) immediately follows domain A and folds as a conserved eight-stranded motif (β1″-β8″), the so-called Greek key topology (7). The active site is located at the interface between domains A and B with Asp248, Glu278, and Asp340 as the catalytic residues (Fig. 4B).

A structure-based homology search in DALI server (34) revealed that the structure most similar to AmyY is the α-amylase AmyB (PDB ID: 3BC9) (5), with a Z-score of 52.8 and a root mean square deviation (RMSD) of 1.4 Å (389 Cα atoms). AmyY also shows structural similarities to other GH13_5 α-amylases, such as BLA (PDB ID: 1BLI; Z-score: 44.6; RMSD: 1.8 Å) (7) and AmyK38 (PDB ID: 1UD2; Z-score: 44.6; RMSD: 2.0 Å) (11). Except that AmyB has an additional N-domain, structures of these four α-amylases are all composed of domains A, B, and C (Fig. 4A). Domain A and domain C are relatively conserved among them, with domain B as the least similar region. Domain B of AmyB, BLA, and AmyK38 all form a small anti-parallel β-barrel with a hole in the interior, but that of AmyY is irregular (Fig. 4A and C). It has been reported that the unfolding of α-amylases is initiated in domain B, and domain B affects the overall structural stability (7, 35, 36).

### Structural basis for the absence of metal ions in AmyY

**Fig 5:**
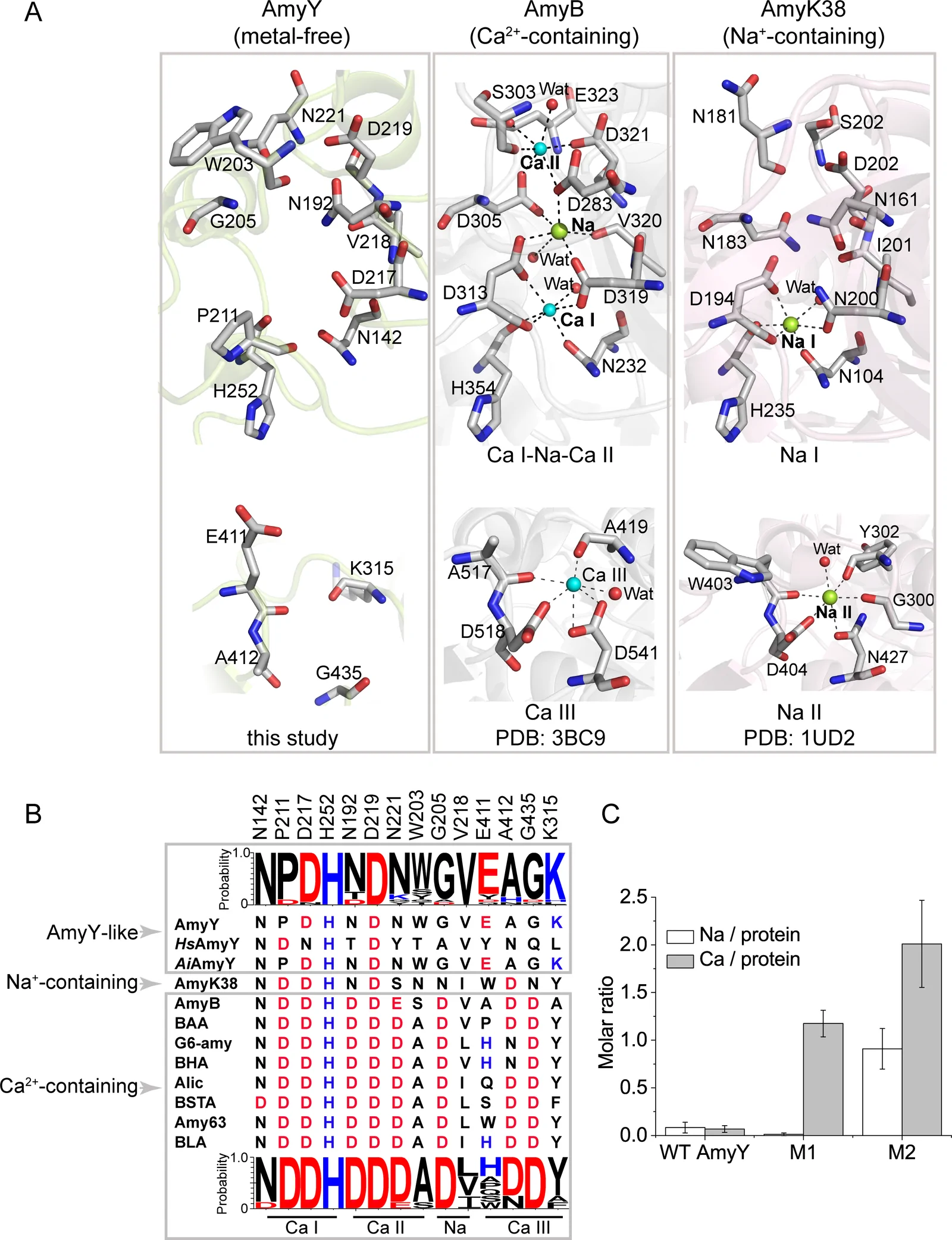
Structural basis for the absence of metal ions in AmyY. (**A**) Comparison of the metal-binding sites (or corresponding sites) in AmyY, AmyB, and AmyK38. Na^+^ and Ca^2+^ are shown as lemon and cyan spheres, respectively; coordination bonds are shown as dashed lines. Wat, water. (**B**) Alignment of the amino acid residues surrounding the metal-binding sites (or corresponding sites) in AmyY and its homologs and structurally characterized α-amylases in GH13_5. Acidic, basic, and neutral amino acid residues are colored, respectively, in red, blue, and black. Sequence logos for AmyY-like proteins and Ca^2+^-containing α-amylases are shown, respectively, above and below the figure. Amino acid residues are numbered according to AmyY. (**C**) ICP-MS determination of Na^+^ and Ca^2+^ contents in WT AmyY and its mutants M1 and M2. Data shown in the graphs are from duplicate experiments (means ± SD).

In all GH13_5 Ca^2+^-containing α-amylases, a Ca^2+^-Na^+^-Ca^2+^ triad (Ca I-Na-Ca II) is located at the interface between domains A and B, and another Ca^2+^ ion (Ca III) between domains A and C, for example, those in AmyB (5) and BLA (7) (Fig. 2 and Fig. 4A). In the structure of the Ca^2+^-free AmyK38, Ca I and Ca III are replaced by two Na^+^ ions, Na I and Na II (11). In contrast, as mentioned above, ICP-MS data indicated that AmyY contains no metal ions, and no significant peaks for metal ions were observed in the electron density map of AmyY. To reveal the structural basis, a detailed structural comparison of AmyY was performed with its closest structural homolog, AmyB (5). Residues Asp313, Asp283, Asp305, Glu323, and Ser303 coordinating the Ca I-Na-Ca II triad of AmyB are replaced, respectively, by Pro211, Asn192, Gly205, Asn221, and Trp203 in AmyY (Fig. 5A). Ala517, Asp518, Ala419, and Asp541 coordinating the Ca III of AmyB are replaced, respectively, by Glu411, Ala412, Lys315, and Gly435 in AmyY. These replacements alter the coordination geometries of the metal-binding sites, leading to the inability of AmyY to bind metal ions. We also noted that predominantly, acidic amino acid residues in AmyB (Asp313, Asp283, Asp305, Glu323, Asp518, and Asp541) are replaced by neutral ones in AmyY (Pro211, Asn192, Gly205, Asn221, Ala412, and Gly435), which may reduce the negative charges and consequently disfavor binding metal cations. Multiple sequence alignment showed that amino acid residues coordinating metal-binding sites in AmyB, especially acidic ones, are conserved in Ca^2+^-containing α-amylases (Fig. 5B). We further mutated the relevant neutral amino acid residues in AmyY to acidic Asp, generating three AmyY mutants: G205D/P211D/N221D (named M1), N192D/G205D/P211D/N221D (named M2), and A412D/G435D. While M1 and M2 were successfully overexpressed in *E. coli*, the expression level of A412D/G435D was too low to be purified. ICP-MS analysis indicated that each M1 molecule binds one Ca²^+^ ion, and each M2 molecule binds two Ca²^+^ ions and one Na^+^ ion (Fig. 5C), suggesting that the mutation restored the ability of AmyY to bind metal ions. These analyses demonstrated that the inability of AmyY to bind metal ions is due to the replacement of acidic amino acid residues by neutral ones. In AmyK38, the replacement of Ca^2+^ by Na^+^ is also due to the substitution of acidic amino acid residues by neutral ones. However, the coordination geometries of the conserved sites of Ca I and Ca III are comparatively well preserved and induce binding Na^+^ (Na I and Na II in AmyK38) rather than Ca^2+^ to both sites (11).

Multiple sequence alignment showed that AmyY homologs exhibit amino acid residue substitutions similar to those in AmyY (Fig. 5B), suggesting that these AmyY homologs may also be unable to bind metal ions. To test our hypothesis, two homologs of AmyY, including *Ai*AmyY (sequence identity to AmyY: 86.4%) from *Arsukibacterium ikkense* and *Hs*AmyY (sequence identity to AmyY: 50.8%) from *Halonatronum saccharophilum* (Fig. 2), were overexpressed in *E. coli* and purified. *Ai*AmyY and *Hs*AmyY exhibited activity on soluble starch with the specific activity of 1,544.6 U/mg and 10,259.6 U/mg, respectively, at 50°C in the glycine-NaOH buffer (pH 10.0). ICP-MS analysis with purified *Ai*AmyY and *Hs*AmyY dissolved in a metal-free Tris-HCl buffer suggested that neither *Ai*AmyY nor *Hs*AmyY contained Ca^2+^, Na^+^, Li^+^, K^+^, Mg^2+^, Fe^2+^, Cu^2+^, or Zn^2+^, similar to AmyY. In addition, AmyA, a characterized α-amylase with 95.1% sequence identity to AmyY, was reported to tolerate 10 mM EDTA and EGTA (22), implying that it may not contain Ca^2+^ ions, unlike canonical α-amylases. Altogether, these data suggest that AmyY and its homologs represent a novel class of metal-free α-amylases in GH13.

### Active site of AmyY

Acarbose, composed of valienamine (VAE), 6-deoxy-D-glucoside (6DG), and a maltose molecule, is a pseudo-tetrasaccharide and commonly used as the inhibitor of α-amylases (5). To gain insights into the active site of AmyY, the crystal structure of AmyY in complex with acarbose (AmyY_ACR_) was solved to be 2.00Å resolution (Table 2). The crystals of AmyY_ACR_ belong to the *P*3_1_21 space group, and two molecules are arranged as a dimer per asymmetric unit. Because AmyY is a monomer in solution (Fig. 3A), the dimerization may be a result of crystal packing. Therefore, chain A of AmyY_ACR_ was used for further analysis. As in the structure of AmyY, the electron density maps corresponding to the CBM20 (residues 494–587) are not visible in AmyY_ACR_.

**Fig 6:**
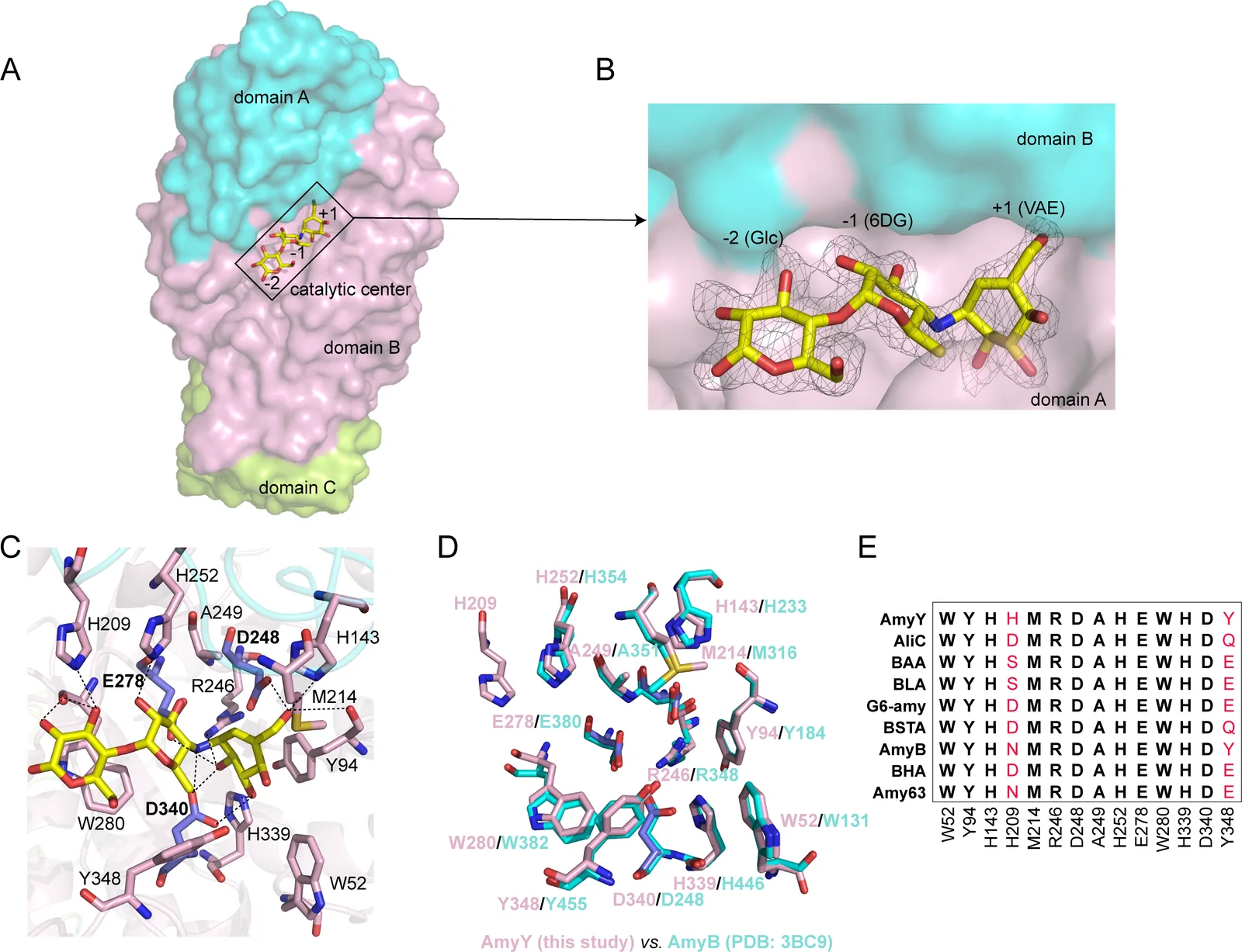
Structural analysis of AmyY in complex with an acarbose derivative. (**A**) Overall structure of AmyY in complex with the acarbose derivative. (**B**) *Fo-Fc* omit map of the acarbose derivative in AmyY_ACR_. The simulated annealing *Fo-Fc* omit map was generated using the Phenix program. The resulting electron density map was contoured at 3σ in gray. The acarbose derivative, composed of valienamine (VAE), 6-deoxy-D-glucoside (6DG), and a glucose molecule (Glc), spans the subsites −2 to +1 of AmyY. (**C**) Key amino acid residues interacting with the acarbose derivative in the active site of AmyY. The acarbose derivative, catalytic residues, and binding residues are shown as yellow, purple, and light pink sticks, respectively. Hydrogen bonds are shown as dashed lines. (**D**) Superposition of substrate-binding pocket of AmyY and its closest structural homolog AmyB (PDB: 3BC9). (**E**) Conservation of the active site residues in AmyY and reported GH13_5 α-amylases with structural data (shown in Fig. 1B). Non-conserved residues are shown in red. Amino acid numbers of AmyY are shown at the bottom.

In AmyY_ACR_, an acarbose-derived trisaccharide, rather than acarbose, was observed at the interface between domains A and B (Fig. 6A). The trisaccharide spans subsites −2 to +1 of AmyY, with the VAE binding to subsite +1, the 6DG to subsite −1, and one glucose molecule to subsite −2 (Fig. 6B). Acarbose derivatives are frequently observed in protein-acarbose complexes, likely produced by transglycosylation reaction (37, 38). Structural analysis indicated that the catalytic residues of AmyY are Asp248 (nucleophile), Glu278 (proton donor), and Asp340 (stabilizing the transition state) (Fig. 6C), which are highly conserved in reported GH13_5 α-amylases (Fig. 6D). Trp52, Tyr94, Arg246, Ala249, His252, Trp280, His339, and Tyr348 from domain A and His143, His209, and Met214 from domain B involve in binding the acarbose derivative in AmyY_ACR_ (Fig. 6C). Among these residues, Tyr94, His143, His209, Arg246, His252, Trp280, and His339 form hydrogen bonds with the acarbose derivative; Trp280 offers a hydrophobic stacking platform for subsite −2; Trp52, Met214, Ala249, and Tyr348 provide hydrophobic forces. Except for His209 and Tyr348, these amino acid residues are highly conserved in other GH13_5 α-amylases (Fig. 6D and E). These analyses indicated that AmyY employs a mechanism for substrate binding and catalysis similar to other GH13_5 α-amylases.

### Engineering of AmyY and assessment of the application potentials of its mutant

AmyY exhibits high activity and tolerance to alkaline pH, salinity, chelating reagents, and surfactants. However, its thermostability is moderate (Fig. 3F). To enhance the thermostability of AmyY, we next set out to engineer AmyY. CBM20 is often located at the C-terminal of α-amylases and facilitates the binding of α-amylases to insoluble raw starch (39). Although AmyY contains a CBM20, it is inactive on raw corn starch (Fig. 3B), suggesting that the CBM20 may be dispensable for the hydrolysis of AmyY. In addition, the absence of CBM20 in AmyY structures (as mentioned above) suggests that the CBM20 is highly flexible and may affect the overall structural stability of AmyY. Therefore, we truncated the CBM20 (Ser501-Phe587) from AmyY and constructed the mutant ΔCBM20. The truncation of CBM20 did not significantly affect the activity of AmyY (2,289.1 ± 117.5 U/mg, 92.1% of WT AmyY). In addition, ΔCBM20 had identical optimum pH (10.0) and temperature (50°C) as WT AmyY.

**Fig 7:**
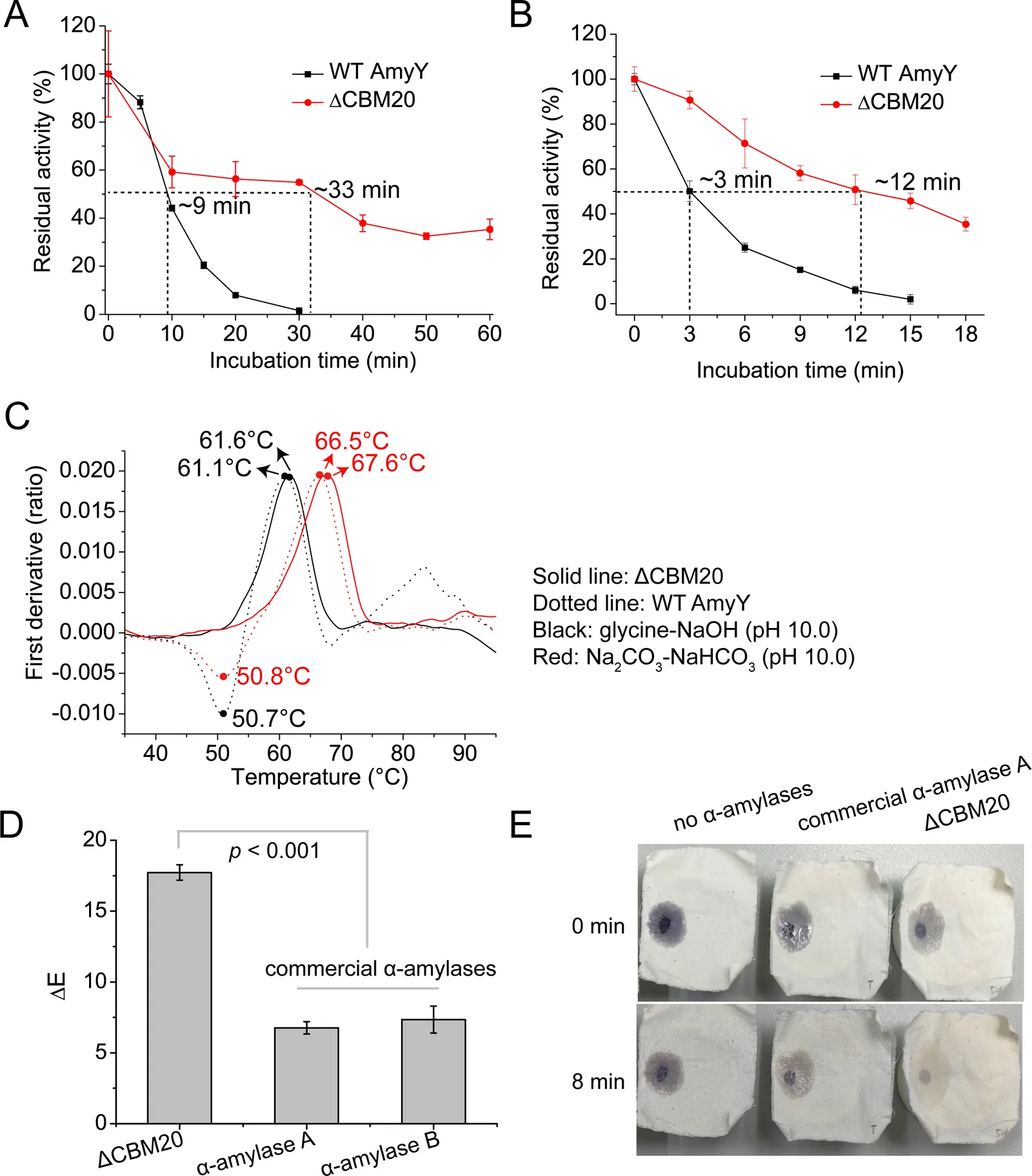
Properties and application potentials of ΔCBM20. (**A and B**) The stability of WT AmyY and ΔCBM20 at 50°C (**A**) and 55°C (**B**). (**C**) *T*_i_ measurements of WT AmyY and ΔCBM20 at pH 10.0 in the glycine-NaOH buffer and the Na_2_CO_3_-NaHCO_3_ buffer. (**D**) Wash performance of ΔCBM20. ΔE represents the difference in whiteness of the fabric before and after 25-min washing in Na_2_CO_3_-NaHCO_3_ buffers (pH 10.0) containing ΔCBM20, commercial α-amylase A, and commercial α-amylase B, respectively. (**E**) Desizing capacity of ΔCBM20. Unscoured cotton fabrics were treated by three distinct solutions: a Na_2_CO_3_-NaHCO_3_ buffer (pH 10.0; as control), one containing ΔCBM20, and one containing commercial α-amylase A. After 20-min treatment, Lugol’s iodine was added, and images at both 0 and 8 min were recorded. Data shown in the graph are from triplicate experiments (means ± SD or representative results).

We further analyzed the stability of ΔCBM20 in glycine-NaOH buffer (pH 10.0) at 50°C and 55°C, separately. The results showed that the half-lives of ΔCBM20 were 33 and 12 min, respectively, at 50°C and 55°C, ~4-fold longer than those of WT AmyY (9 min at 50°C and 3 min at 55°C) (Fig. 7A and B), indicating that ΔCBM20 has a higher thermostability than AmyY. To further quantify the stability, the unfolding inflection temperatures (*T*_i_) of ΔCBM20 and WT AmyY at pH 10.0 were determined using Tycho NT.6. WT AmyY had two *T*_i_ values in both the glycine-NaOH buffer (50.7°C and 61.1°C) and the Na_2_CO_3_-NaHCO_3_ buffer (50.8°C and 66.5°C) (Fig. 7C), suggesting that AmyY has at least two stages in its denaturation process. The first stage is the unfolding of CBM20, and the second is that of its catalytic module. ΔCBM20 retains only the second stage with a 0.5°C (in glycine-NaOH) or 1.1°C (in Na_2_CO_3_-NaHCO_3_) higher *T*_i_ value (Fig. 7C). These results suggested that the thermal unfolding of AmyY is likely initiated in its CBM20, and the CBM20 truncation improved the overall structural stability of AmyY.

The application potentials of ΔCBM20 in the detergent and textile industries, where alkaline α-amylases are of great interest, were evaluated by wash performance analysis and desizing capacity tests. The wash performance analysis showed that in the Na_2_CO_3_-NaHCO_3_ buffer (pH 10.0), ΔCBM20 demonstrated significantly superior starch stain removal capacity compared with α-amylases A and B, two commercial detergent additives (Fig. 7D; the specific commercial identities of α-amylases A and B are not disclosed at the request of the supplier). In the desizing assay, a markedly lighter iodine staining color was observed on the ΔCBM20-treated fabric at both 0 min and 8 min (Fig. 7E), demonstrating a significantly better desizing capacity of ΔCBM20 than commercial α-amylase A. These results underscore the application potentials of ΔCBM20 in the detergent and textile industries.

## DISCUSSION

Ca²⁺^+^ ions are generally present at the interface between domains A and B and between domains A and C in GH13 α-amylases (Fig. 1 and Fig. 4A). The activity of Ca^2+^-containing α-amylases is often dependent on exogenous Ca^2+^. For example, AmyB exhibits optimum activity at ≥0.2 mM Ca^2+^ and has <20% activity in the absence of Ca^2+^ (5). Moreover, Ca^2+^ ions in α-amylases are often readily removed by chelating reagents, resulting in reduced activity and stability (5, 11, 12, 29). Here, we studied an alkaline α-amylase, AmyY, which contains a catalytic module (composed of domains A, B, and C) and a CBM20 domain. In the structure of AmyY lacking the CBM20 domain, no significant peaks for metal ions were observed, particularly at positions where Ca²^+^ ions are typically found in α-amylases. ICP-MS analysis of the full-length AmyY revealed the absence not only of Ca²^+^ but also of other metal cations commonly present in protein structures (e.g., Na^+^, Li^+^, K^+^, Mg²^+^, Fe²^+^, Cu²^+^, and Zn²^+^). These results indicate that neither the catalytic module nor the CBM20 domain of AmyY binds metal ions. Thus, AmyY represents a novel metal-free α-amylase in GH13. Site-directed mutation analysis indicated that replacements of acidic amino acid residues by neutral ones alter the coordination geometries and reduce the negative charges of metal-binding sites, leading to the inability of AmyY to bind metal ions. Similar replacements were also observed in AmyY homologs, and two homologs were demonstrated to be metal-free by ICP-MS. Although some metal-free α-amylases have been reported that share the same catalytic residues (Asp, Glu, Asp) and catalytic mechanism with AmyY (13–17), they are atypical α-amylases. Their substrate-binding architectures differ from those of typical α-amylases, and they preferentially degrade starch-related substrates (e.g., cyclodextrin) rather than starch (13–17). Therefore, in this study, we biochemically and structurally revealed a class of typical α-amylases containing no metals in GH13, represented by AmyY.

AmyY and its homologs are primarily from alkaline and hypersaline environments, such as soda lakes (Fig. 2), where the bioavailability of Ca²⁺^+^ is extremely low—a direct consequence of Ca²⁺^+^ precipitation driven by high carbonate concentrations (40). Therefore, AmyY likely represents an evolutionary adaptation to this metal-limited environment. Compared with metal-containing α-amylases, AmyY exhibited a specific activity of 2,485 U/mg (158,294 U/µmol) on soluble starch, higher than that of BLA (PDB: 1BLI; 117,000 U/µmol and 2,000 U/mg) (41), comparable to that of PFA (PDB: 1MWO; 195,000 U/µmol and 3900 U/mg) (41), and slightly lower but within the same order of magnitude as those of LAMY (PDB: 2DIE; 265,000 U/µmol and 5000 U/mg) (42) and AmyK38 (PDB: 1UD2; 257,757 U/µmol and 4678 U/mg) (12). Thus, it seems that the absence of metal ions in AmyY does not affect the enzyme activity.

Compared with α-amylases containing metal ions, metal-free α-amylases, such as AmyY and its homologs, may have advantages in industrial applications. First, they do not require the addition of any metal ion for full catalytic activity, which can maximize industrial efficiency. Second, alkaline α-amylases are widely used in detergents, where chelating reagents are usually contained. The metal-free α-amylases that resist chelating reagents are ideal candidates for the detergent industry.

CBMs are frequently accessory domains of glycoside hydrolases, primarily involved in substrate binding rather than enzyme catalysis, thereby enhancing enzymatic activity. Furthermore, CBMs can affect the thermostability and substrate specificity of enzymes (43). However, not all CBMs confer advantages to enzyme performances. For example, the CBM truncation of alkaline α-amylase AmyK from *Alkalimonas amylolytica* improves its activity and oxidative stability (25). Similarly, another report demonstrates that the activity and thermostability of xylanase XynA from *Caldicellulosiruptor* sp. are increased via CBM truncation (44). Consequently, CBM truncation presents a viable strategy in protein engineering. Here, structural analysis revealed that the CBM20 of AmyY exhibits conformational flexibility. After CBM20 truncation, the thermostability of AmyY was enhanced by approximately fourfold. *T*_i_ measurements suggested that the thermal unfolding of AmyY is initiated in its CBM20 (Fig. 7C). CBM20 truncation likely reduced unstable regions and increased the overall structural compactness, thereby improving the thermostability of AmyY. Compared with the commercial α-amylases, the CBM20 truncation mutant displayed remarkable wash performance and desizing capability at pH 10.0 (Fig. 7D and E), demonstrating its application potentials in the detergent and textile industries where alkaline α-amylases are of great interest.

## MATERIALS AND METHODS

### Materials and bacterial strains

Soluble starch, amylose, and amylopectin were purchased from Sigma (USA). Corn starch was purchased from a supermarket (China). G2 was purchased from Solarbio (China), and other malto-oligosaccharides (from G3 to G6) were purchased from Megazyme (Ireland). *Alkalimonas* sp. NCh-2 was isolated from an alkaline hot spring (25.465°N, 100.781°E) in Xiangyun County, Yunnan Province, China. The medium (pH 10.0) used contained 1.0% (wt/vol) soluble starch, 1.0% (wt/vol) Na_2_CO_3_, 0.6% (wt/vol) NaHCO_3_, 0.25% (wt/vol) NaOH, 0.1% (wt/vol) K_2_HPO_4_, 0.02% (wt/vol) MgSO_4_·7H_2_O, 0.05% (wt/vol) NH_4_Cl, and 0.1% (vol/vol) vitamin solution. The compositions of the vitamin solution are as described previously (45). The cultivation temperature was 37°C. *E. coli* DH5α and *E. coli* BL21(DE3) (Vazyme, China) were used for gene construction and protein production, respectively.

### Gene cloning and mutagenesis

After deleting the predicted signal peptides, genes encoding AmyY and its CBM20-truncation mutants were cloned from the genomic DNA of *Alkalimonas* sp. NCh-2 by PCR amplification. These genes were inserted into the NdeI/XhoI restriction sites of the pET22b vector with a C-terminal His-tag using an In-Fusion HD cloning kit (TaKaRa, Japan). M1 and M2 were constructed by QuikChange (46) using the plasmid pET22b-AmyY as the template. Genes encoding *Ai*AmyY (WP_046556027.1) from *Arsukibacterium ikkense* and *Hs*AmyY (WP_084804421.1) from *Halonatronum saccharophilum* without the predicted signal peptide were synthesized at Beijing Tsingke (China) and inserted into pET22b.

### Protein production and purification

The constructed plasmids were transferred into *E*. coli BL21(DE3). The cells were cultured in the Luria-Bertani (LB) medium containing 100 µg/mL ampicillin at 37°C to an OD_600_ of 0.6–0.8. The cells were then induced by 100 µM isopropyl-β-D-thiogalactopyranoside (IPTG) and incubated at 18°C for 16 h. Next, the cells were harvested (8000 *g* for 10 min at 4°C) and crushed by a cell crusher in a lysis buffer (50 mM Tris-HCl, 100 mM NaCl, 0.5% glycerol, pH 8.0). Purification of the proteins was performed by using the nickel affinity chromatography on Ni^2+^-nitrilotriacetic acid (NTA) columns and followed by desalination on PD-10 desalting columns with 10 mM Tris-HCl (pH 8.0) containing 100 mM NaCl. These columns were all purchased from GE Healthcare (USA) and operated according to the manufacturer’s instructions. Protein purities were analyzed by SDS-PAGE. Protein concentrations were determined by a bicinchoninic acid protein assay kit (Thermo, USA) with bovine serum albumin as the standard.

### Enzyme assay

Unless otherwise stated, enzyme activities were determined by the 3,5-dinitrosalicylic acid (DNS) method (47) at 50°C in glycine-NaOH buffer (50 mM, pH 10.0). The standard reaction mixture contained 10 µl AmyY (or its mutants [0.8–2.0 µg/mL]) and 90 µL soluble starch (10 mg/mL). After incubation for 10 min, reactions were terminated by adding 100 µl DNS. Next, samples were incubated at 100°C for 5 min, and then their OD_550_ was measured. The standard curve was generated with glucose (0–1.0 mg/mL). One unit of enzyme activity (U) was defined as the amount of enzyme required to release 1 µmol glucose equivalents per min.

### Biochemical characterization

The substrate specificity of AmyY was determined with soluble starch, amylopectin, amylose, and corn starch as the substrate, separately, at a concentration of 10 mg/mL. Other characteristics were studied with soluble starch from potato (ACS reagent grade, Sigma Product No. S9765) as the substrate, which was prepared with glycine-NaOH buffer (50 mM, pH 10.0) and dissolved by microwave heating. The effect of temperature on AmyY activity was determined from 20°C to 70°C. For thermostability assay, AmyY or its mutants were incubated separately at 45°C, 50°C, and 55°C for 0–1 h, and at an interval of 10 min, residual activities were determined at 50°C and pH 10.0. The half-life was defined as the time required for the enzyme activity to be reduced by 50%. The effect of pH on AmyY activity was determined at 50°C using Britton-Robinson buffers (50 mM) with pH values from 6.0 to 12.0. For the pH stability assay, AmyY was incubated in Britton-Robinson buffers (50 mM) from pH 6.0 to 12.0, separately, at 40°C for 3 h, and residual activities were determined at 50°C and pH 10.0. The effect of the NaCl on AmyY activity was assayed at 50°C and pH 10.0 by the addition of NaCl from 0 M to 5 M. For the NaCl tolerance assay, AmyY was incubated in the presence of 0–5 M NaCl at 4°C for 24 h in glycine-NaOH buffer (50 mM, pH 10.0), and residual activities were determined at 50°C and pH 10.0. To determine the effect of metal ions and chemical reagents on the activity of AmyY, AmyY was pre-incubated in glycine-NaOH buffer (50 mM, pH 10.0) containing one metal ion or chemical reagent at a final concentration of 10 mM or 100 mM at 4°C for 1 h, and then the residual activities were determined at 50°C and pH 10.0.

### Mode of action analysis

Reaction mixtures containing 270 µL soluble starch (10 mg/mL) and 30 µL AmyY (50 µg/mL) in glycine-NaOH buffer (50 mM, pH 10.0) were incubated at 30°C. At 0, 1, 6, 12, and 24 h separately, the reaction was terminated by boiling for 10 min. The products were analyzed using high-performance liquid chromatography (HPLC) equipped with an evaporative light-scattering detector (ELSD). The column used was a Superdex 30 Increase 10/300 GL column (GE Healthcare, USA). The eluent was ultrapure water at a flow rate of 0.3 mL/min. A mixture of glucose and malto-oligosaccharides (from G2 to G6) was used as the standard.

### Protein crystallization, data collection, and structure determination

Protein crystallization was performed using the hanging-drop vapor-diffusion method at 18°C. Crystals of AmyY were obtained in the buffer containing 0.2 M sodium iodide, 0.1 M Bis-Tris propane (pH 7.5), and 20% (wt/vol) PEG 3350. To obtain crystals of AmyY in complex with acarbose, 10 mg/mL AmyY and 1 mg/mL acarbose (Solarbio, China) were incubated at 4°C for 30 min before crystallization. Crystals of the complex were obtained in the buffer containing 0.2 M sodium fluoride, 0.1 M Bis-Tris propane (pH 7.5), and 20% (wt/vol) PEG 3350. X-ray diffraction data were collected on the BL18U1 beamline at the Shanghai Synchrotron Radiation Facility. The initial diffraction data sets were processed by the X-ray Detector Software. With the predicted structure of AmyY by AlphaFold2 (48) as a search model, structures of AmyY and its complex with acarbose were solved by molecular replacement using CCP4. Refinements of these structures were performed using Coot and Phenix. All structural figures were processed using PyMOL. Protein-ligand interactions were analyzed by Ligplot (49).

### DLS analysis

DLS assay was performed on a DynaPro NanoStar (Wyatt Technology, USA) at 25°C using 10 µL AmyY (2.0 mg/mL) in 10 mM Tris-HCl (pH 8.0) containing 100 mM NaCl. Data were analyzed with Dynamics 7.1.0.

### Detection of metal ions

Metal ions in AmyY and its homologs (*Ai*AmyY and *Hs*AmyY) were quantified by ICP-MS. To eliminate the effect of exogenous metal ions on ICP-MS data, the metal-free buffer, 50 mM Tris-HCl (pH 8.0), was used in the desalination of AmyY on PD-10 desalting columns before ICP-MS. A mixture of 1 mL protein solution (at least 20 mg/mL) and 5 mL nitric acid was incubated at 130°C overnight. The sample was then diluted to 12 mL with ultrapure water, and metal ions Na^+^, K^+^, Li^+^, Ca^2+^, and Zn^2+^ in the sample were quantified on an ICP-MS NexION 1000G (PerkinElmer, USA). Tris-HCl buffer (50 mM; pH 8.0) was used as the control.

### *T*_i_ measurement

*T*_i_ values of AmyY and ΔCBM20 were determined using Tycho NT.6 (NanoTemper, Germany) (50). AmyY and ΔCBM20 were diluted to 0.1 mg/mL with glycine-NaOH and Na_2_CO_3_-NaHCO_3_ buffer (pH 10.0), separately. Intensities of intrinsic fluorescence at 330 nm and 350 nm were recorded with increasing temperatures from 35°C to 95°C at a rate of 3°C/min. *T*_i_ values were calculated from the temperature-dependent change in the ratio of fluorescence signals (350 nm/330 nm).

### Analyses of potential industrial applications

The wash performance was conducted using starch-soiled test fabrics C-S-28 (3 cm × 3 cm) according to the method previously reported (51) with some modification. Before washing, the whiteness (values from 0 [black] to 100 [white]) of both the front (A0) and back sides (B0) of each fabric was detected by a brightness meter. Then, the fabrics were randomly separated into three groups and were dipped into separate Na_2_CO_3_-NaHCO_3_ buffers (pH 10.0, 1,000 mL) containing ΔCBM20, commercial α-amylase A (DuPont, USA), and commercial α-amylase B (Novozymes, Denmark) at the same concentration. After stirring at 30°C and 40 rpm for 25 min, the fabrics were rinsed twice with ultrapure water and air dried. The stain removal effect was determined according to the formula ΔE = [(A1 − A0) + (B1 − B0)] / 2, where A1 (the front side) and B1 (the back side) represent the whiteness of fabrics after washing. In the desizing assay, the unscoured cotton fabrics (5 cm × 5 cm) were randomly separated into three groups and separately put into 10 mL Na_2_CO_3_-NaHCO_3_ buffers (pH 10.0), 10 mL Na_2_CO_3_-NaHCO_3_ buffers (pH 10.0) + 0.2 mL ΔCBM20, and 10 mL Na_2_CO_3_-NaHCO_3_ buffers (pH 10.0) + 0.2 mL commercial α-amylase A (equivalent concentration to ΔCBM20). After 20-min incubation, the fabrics were rinsed five times with ultrapure water, drying at 45°C, and colored using Lugol’s iodine. Images were recorded at both 0 and 8 min after the addition of Lugol’s iodine.

### Bioinformatics

The signal peptide was predicted by SignalP 5.0 (52). Domain analysis was performed in the NCBI conserved domain database (53). AmyY homologs were searched using BlastP in the NCBI-nr (non-redundant protein sequences) database with cut-off values of sequence identity > 50% and coverage > 75%. Multiple sequence alignments were carried out with CLC Sequence Viewer 6. Sequence logos were drawn with WebLogo 3 (http://weblogo.threeplusone.com).

## Data Availability

The genomic data of *Alkalimonas* sp. NCh-2 and the amino acid sequence of AmyY are available in NCBI under accession numbers GCA_039631345.1 and MEN3159421.1, respectively. The atomic coordinates and structure factors of AmyY and its complex with acarbose have been deposited in PDB under accession numbers 24ZP and 24ZQ, respectively.

## References

[1] Sindhu R, Binod P, Madhavan A, Beevi US, Mathew AK, Abraham A, Pandey A, Kumar V. 2017. Molecular improvements in microbial α-amylases for enhanced stability and catalytic efficiency. Bioresour Technol 245:1740–1748. doi:10.1016/j.biortech.2017.04.098 28478894

[2] Kikani BA, Singh SP. 2022. Amylases from thermophilic bacteria: structure and function relationship. Crit Rev Biotechnol 42:325–341. doi:10.1080/07388551.2021.1940089 34420464

[3] Drula E, Garron ML, Dogan S, Lombard V, Henrissat B, Terrapon N. 2022. The carbohydrate-active enzyme database: functions and literature. Nucleic Acids Res 50:D571–D577. doi:10.1093/nar/gkab1045 PMC8728194 34850161

[4] Stam MR, Danchin EGJ, Rancurel C, Coutinho PM, Henrissat B. 2006. Dividing the large glycoside hydrolase family 13 into subfamilies: towards improved functional annotations of alpha-amylase-related proteins. Protein Eng Des Sel 19:555–562. doi:10.1093/protein/gzl044 17085431

[5] Tan TC, Mijts BN, Swaminathan K, Patel BKC, Divne C. 2008. Crystal structure of the polyextremophilic alpha-amylase AmyB from *Halothermothrix orenii*: details of a productive enzyme-substrate complex and an N domain with a role in binding raw starch. J Mol Biol 378:852–870. doi:10.1016/j.jmb.2008.02.041 18387632

[6] Rodríguez-Sanoja R, Oviedo N, Sánchez S. 2005. Microbial starch-binding domain. Curr Opin Microbiol 8:260–267. doi:10.1016/j.mib.2005.04.013 15939348

[7] Declerck N, Machius M, Wiegand G, Huber R, Gaillardin C. 2000. Probing structural determinants specifying high thermostability in *Bacillus* licheniformis alpha-amylase. J Mol Biol 301:1041–1057. doi:10.1006/jmbi.2000.4025 10966804

[8] Linden A, Mayans O, Meyer-Klaucke W, Antranikian G, Wilmanns M. 2003. Differential regulation of a hyperthermophilic alpha-amylase with a novel (Ca,Zn) two-metal center by zinc. J Biol Chem 278:9875–9884. doi:10.1074/jbc.M211339200 12482867

[9] Kohli I, Joshi NC, Varma A. 2020. Production, purification and applications of raw starch degrading and calcium-independent α-amylase from soil rich in extremophile. Int J Biol Macromol 162:873–881. doi:10.1016/j.ijbiomac.2020.06.160 32565305

[10] Haki GD, Rakshit SK. 2003. Developments in industrially important thermostable enzymes: a review. Bioresour Technol 89:17–34. doi:10.1016/s0960-8524(03)00033-6 12676497

[11] Nonaka T, Fujihashi M, Kita A, Hagihara H, Ozaki K, Ito S, Miki K. 2003. Crystal structure of calcium-free alpha-amylase from *Bacillus* sp. strain KSM-K38 (AmyK38) and its sodium ion binding sites. J Biol Chem 278:24818–24824. doi:10.1074/jbc.M212763200 12719434

[12] Hagihara H, Hayashi Y, Endo K, Igarashi K, Ozawa T, Kawai S, Ozaki K, Ito S. 2001. Deduced amino-acid sequence of a calcium-free alpha-amylase from a strain of *Bacillus*: implications from molecular modeling of high oxidation stability and chelator resistance of the enzyme. Eur J Biochem 268:3974–3982. doi:10.1046/j.1432-1327.2001.02308.x 11453991

[13] Park JT, Song HN, Jung TY, Lee MH, Park SG, Woo EJ, Park KH. 2013. A novel domain arrangement in a monomeric cyclodextrin-hydrolyzing enzyme from the hyperthermophile *Pyrococcus furiosus*. Biochim Biophys Acta 1834:380–386. doi:10.1016/j.bbapap.2012.08.001 22902546

[14] Jung T-Y, Li D, Park J-T, Yoon S-M, Tran PL, Oh B-H, Janeček Š, Park SG, Woo E-J, Park K-H. 2012. Association of novel domain in active site of archaic hyperthermophilic maltogenic amylase from *Staphylothermus marinus*. J Biol Chem 287:7979–7989. doi:10.1074/jbc.M111.304774 PMC3318708 22223643

[15] Lee HS, Kim MS, Cho HS, Kim JI, Kim TJ, Choi JH, Park C, Lee HS, Oh BH, Park KH. 2002. Cyclomaltodextrinase, neopullulanase, and maltogenic amylase are nearly indistinguishable from each other. J Biol Chem 277:21891–21897. doi:10.1074/jbc.M201623200 11923309

[16] Feese MD, Kato Y, Tamada T, Kato M, Komeda T, Miura Y, Hirose M, Hondo K, Kobayashi K, Kuroki R. 2000. Crystal structure of glycosyltrehalose trehalohydrolase from the hyperthermophilic archaeum *Sulfolobus solfataricus*. J Mol Biol 301:451–464. doi:10.1006/jmbi.2000.3977 10926520

[17] Jun SY, Kim JS, Choi KH, Cha J, Ha NC. 2013. Structure of a novel α-amylase AmyB from *Thermotoga neapolitana* that produces maltose from the nonreducing end of polysaccharides. Acta Crystallogr D 69:442–450. doi:10.1107/S0907444912049219 23519419

[18] Saxena RK, Dutt K, Agarwal L, Nayyar P. 2007. A highly thermostable and alkaline amylase from a *Bacillus* sp. PN5. Bioresour Technol 98:260–265. doi:10.1016/j.biortech.2006.01.016 16524725

[19] Medda S, Chandra AK. 1980. New strains of *Bacillus licheniformis* and *Bacillus coagulans* producing thermostable alpha-amylase active at alkaline pH. J Appl Bacteriol 48:47–58. doi:10.1111/j.1365-2672.1980.tb05205.x 6154683

[20] Montor-Antonio JJ, Hernández-Heredia S, Ávila-Fernández Á, Olvera C, Sachman-Ruiz B, Del Moral S. 2017. Effect of differential processing of the native and recombinant α-amylase from *Bacillus amyloliquefaciens* JJC33M on specificity and enzyme properties. 3 Biotech 7:336. doi:10.1007/s13205-017-0954-8 PMC5605471 28955633

[21] Priyadarshini S, Pradhan SK, Ray P. 2020. Production, characterization and application of thermostable, alkaline α-amylase (AA11) from *Bacillus cereus* strain SP-CH11 isolated from Chilika lake. Int J Biol Macromol 145:804–812. doi:10.1016/j.ijbiomac.2019.11.149 31758985

[22] Wang N, Zhang YH, Wang QH, Liu J, Wang HL, Xue YF, Ma YH. 2006. Gene cloning and characterization of a novel alpha-amylase from alkaliphilic *Alkalimonas amylolytica*. Biotechnol J 1:1258–1265. doi:10.1002/biot.200600098 17068753

[23] Yang G, Yao H, Mozzicafreddo M, Ballarini P, Pucciarelli S, Miceli C. 2017. Rational engineering of a cold-adapted α-Amylase from the antarctic ciliate *Euplotes focardii* for simultaneous improvement of thermostability and catalytic activity. Appl Environ Microbiol 83:e00449-17. doi:10.1128/AEM.00449-17 PMC5478988 28455329

[24] Yang HQ, Liu L, Wang MX, Li JH, Wang NS, Du GC, Chen J. 2012. Structure-based engineering of methionine residues in the catalytic cores of alkaline amylase from *Alkalimonas amylolytica* for improved oxidative stability. Appl Environ Microbiol 78:7519–7526. doi:10.1128/AEM.01307-12 PMC3485717 22865059

[25] Yang H, Liu L, Shin H, Chen RR, Li J, Du G, Chen J. 2013. Integrating terminal truncation and oligopeptide fusion for a novel protein engineering strategy to improve specific activity and catalytic efficiency: alkaline α-amylase as a case study. Appl Environ Microbiol 79:6429–6438. doi:10.1128/AEM.02087-13 PMC3811186 23956385

[26] Yang H, Lu X, Liu L, Li J, Shin H, Chen RR, Du G, Chen J. 2013. Fusion of an oligopeptide to the N terminus of an alkaline α-amylase from *Alkalimonas amylolytica* simultaneously improves the enzyme’s catalytic efficiency, thermal stability, and resistance to oxidation. Appl Environ Microbiol 79:3049–3058. doi:10.1128/AEM.03785-12 PMC3623161 23455344

[27] Allala F, Bouacem K, Boucherba N, Azzouz Z, Mechri S, Sahnoun M, Benallaoua S, Hacene H, Jaouadi B, Bouanane-Darenfed A. 2019. Purification, biochemical, and molecular characterization of a novel extracellular thermostable and alkaline α-amylase from *Tepidimonas fonticaldi* strain HB23. Int J Biol Macromol 132:558–574. doi:10.1016/j.ijbiomac.2019.03.201 30928371

[28] Priyadarshini S, Ray P. 2019. Exploration of detergent-stable alkaline α-amylase AA7 from *Bacillus* SP strain SP-CH7 isolated from Chilika lake. Int J Biol Macromol 140:825–832. doi:10.1016/j.ijbiomac.2019.08.006 31401271

[29] Lu ZH, Wang QH, Jiang SJ, Zhang GM, Ma YH. 2016. Truncation of the unique N-terminal domain improved the thermos-stability and specific activity of alkaline α-amylase Amy703. Sci Rep 6:22465. doi:10.1038/srep22465 PMC4772547 26926401

[30] Zhou GL, Jin M, Cai YP, Zeng RY. 2015. Characterization of a thermostable and alkali-stable α-amylase from deep-sea bacterium *Flammeovirga pacifica*. Int J Biol Macromol 80:676–682. doi:10.1016/j.ijbiomac.2015.07.042 26210035

[31] Hmidet N, Bayoudh A, Berrin JG, Kanoun S, Juge N, Nasri M. 2008. Purification and biochemical characterization of a novel α-amylase from *Bacillus licheniformis* NH1: cloning, nucleotide sequence and expression of amyN gene in *Escherichia coli*. Process Biochem 43:499–510. doi:10.1016/j.procbio.2008.01.017

[32] Yano JK, Poulos TL. 2003. New understandings of thermostable and peizostable enzymes. Curr Opin Biotech 14:360–365. doi:10.1016/s0958-1669(03)00075-2 12943843

[33] Gerlt JA, Raushel FM. 2003. Evolution of function in (β/α)-barrel enzymes. Curr Opin Chem Biol 7:252–264. doi:10.1016/s1367-5931(03)00019-x 12714059

[34] Holm L, Rosenström P. 2010. Dali server: conservation mapping in 3D. Nucleic Acids Res 38:W545–W549. doi:10.1093/nar/gkq366 PMC2896194 20457744

[35] Joyet P, Declerck N, Gaillardin C. 1992. Hyperthermostable variants of a highly thermostable α-amylase. Biotechnology (N Y) 10:1579–1583. doi:10.1038/nbt1292-1579 1369206

[36] Liao M, Feng SH, Liu XQ, Xu GS, Li SC, Bai YG, Luo HY, Yao B, Wang HB, Tu T. 2024. Novel Insights into enzymatic thermostability: the “Short Board” theory and zero-shot hamiltonian model. Adv Sci (Weinh) 11:e2402441. doi:10.1002/advs.202402441 PMC11615740 39308285

[37] Rozeboom HJ, Yu SK, Madrid S, Kalk KH, Zhang R, Dijkstra BW. 2013. Crystal structure of α-1,4-glucan lyase, a unique glycoside hydrolase family member with a novel catalytic mechanism. J Biol Chem 288:26764–26774. doi:10.1074/jbc.M113.485896 PMC3772222 23902768

[38] Brzozowski AM, Lawson DM, Turkenburg JP, Bisgaard-Frantzen H, Svendsen A, Borchert TV, Dauter Z, Wilson KS, Davies GJ. 2000. Structural analysis of a chimeric bacterial α-amylase: high-resolution analysis of native and ligand complexes. Biochemistry 39:9099–9107. doi:10.1021/bi0000317 10924103

[39] Ngo ST, Tran-Le PD, Ho GT, Le LQ, Bui LM, Vu BK, Thu Phung HT, Nguyen H-D, Vo T-S, Vu VV. 2019. Interaction of carbohydrate binding module 20 with starch substrates. RSC Adv 9:24833–24842. doi:10.1039/C9RA01981B PMC9069913 35528656

[40] Sorokin DY, Banciu HL, Muyzer G. 2015. Functional microbiology of soda lakes. Curr Opin Microbiol 25:88–96. doi:10.1016/j.mib.2015.05.004 26025021

[41] Dong GQ, Vieille C, Savchenko A, Zeikus JG. 1997. Cloning, sequencing, and expression of the gene encoding extracellular alpha-amylase from *Pyrococcus furiosus* and biochemical characterization of the recombinant enzyme. Appl Environ Microbiol 63:3569–3576. doi:10.1128/aem.63.9.3569-3576.1997 PMC168662 9293008

[42] Igarashi K, Hatada Y, Hagihara H, Saeki K, Takaiwa M, Uemura T, Ara K, Ozaki K, Kawai S, Kobayashi T, Ito S. 1998. Enzymatic properties of a novel liquefying alpha-amylase from an alkaliphilic *Bacillus* isolate and entire nucleotide and amino acid sequences. Appl Environ Microbiol 64:3282–3289. doi:10.1128/AEM.64.9.3282-3289.1998 PMC106722 9726872

[43] Hamouda HI, Fan YX, Abdalla M, Su H, Lu M, Li FL. 2023. Distinct roles of carbohydrate-binding modules in multidomain β-1,3-1,4-glucanase on polysaccharide degradation. Appl Microbiol Biotechnol 107:1751–1764. doi:10.1007/s00253-023-12416-4 36800030

[44] Meng DD, Ying Y, Chen XH, Lu M, Ning K, Wang LS, Li FL. 2015. Distinct roles for carbohydrate-binding modules of glycoside hydrolase 10 (GH10) and GH11 xylanases from *Caldicellulosiruptor* sp. strain F32 in thermostability and catalytic efficiency. Appl Environ Microbiol 81:2006–2014. doi:10.1128/AEM.03677-14 PMC4345376 25576604

[45] Sun HN, Yu CM, Fu HH, Wang P, Fang ZG, Zhang YZ, Chen XL, Zhao F. 2021. Diversity of marine 1,3-xylan-utilizing bacteria and characters of their extracellular 1,3-xylanases. Front Microbiol 12:721422. doi:10.3389/fmicb.2021.721422 PMC8517272 34659148

[46] Liu HT, Naismith JH. 2008. An efficient one-step site-directed deletion, insertion, single and multiple-site plasmid mutagenesis protocol. BMC Biotechnol 8:91. doi:10.1186/1472-6750-8-91 PMC2629768 19055817

[47] Miller GL, Blum R, Glennon WE, Burton AL. 1960. Measurement of carboxymethylcellulase activity. Anal Biochem 1:127–132. doi:10.1016/0003-2697(60)90004-X

[48] Jumper J, Evans R, Pritzel A, Green T, Figurnov M, Ronneberger O, Tunyasuvunakool K, Bates R, Žídek A, Potapenko A, et al.. 2021. Highly accurate protein structure prediction with AlphaFold. Nature 596:583–589. doi:10.1038/s41586-021-03819-2 PMC8371605 34265844

[49] Wallace AC, Laskowski RA, Thornton JM. 1995. LIGPLOT: a program to generate schematic diagrams of protein-ligand interactions. Protein Eng 8:127–134. doi:10.1093/protein/8.2.127 7630882

[50] Nosrati M, Dey D, Mehrani A, Strassler SE, Zelinskaya N, Hoffer ED, Stagg SM, Dunham CM, Conn GL. 2019. Functionally critical residues in the aminoglycoside resistance-associated methyltransferase RmtC play distinct roles in 30S substrate recognition. J Biol Chem 294:17642–17653. doi:10.1074/jbc.RA119.011181 PMC6873201 31594862

[51] Wu YM, Huang S, Liang XB, Han P, Liu YC. 2023. Characterization of a novel detergent-resistant type I pullulanase from *Bacillus megaterium* Y103 and its application in laundry detergent. Prep Biochem Biotechnol 53:683–689. doi:10.1080/10826068.2022.2134890 36271878

[52] Almagro Armenteros JJ, Tsirigos KD, Sønderby CK, Petersen TN, Winther O, Brunak S, von Heijne G, Nielsen H. 2019. SignalP 5.0 improves signal peptide predictions using deep neural networks. Nat Biotechnol 37:420–423. doi:10.1038/s41587-019-0036-z 30778233

[53] Lu SN, Wang JY, Chitsaz F, Derbyshire MK, Geer RC, Gonzales NR, Gwadz M, Hurwitz DI, Marchler GH, Song JS, Thanki N, Yamashita RA, Yang MZ, Zhang DC, Zheng CJ, Lanczycki CJ, Marchler-Bauer A. 2020. CDD/SPARCLE: the conserved domain database in 2020. Nucleic Acids Res 48:D265–D268. doi:10.1093/nar/gkz991 PMC6943070 31777944

